# Neuroimaging Insights: Kava’s (*Piper methysticum*) Effect on Dorsal Anterior Cingulate Cortex GABA in Generalized Anxiety Disorder

**DOI:** 10.3390/nu15214586

**Published:** 2023-10-28

**Authors:** Karen Savage, Jerome Sarris, Matthew Hughes, Chad A. Bousman, Susan Rossell, Andrew Scholey, Con Stough, Chao Suo

**Affiliations:** 1 Centre for Human Psychopharmacology, Swinburne University of Technology, 427-451 Burwood Road, Melbourne 3122, Australia; 2 Florey Institute of Neuroscience and Mental Health, Melbourne University, Melbourne 3121, Australia; 3 NICM Health Research Institute, Western Sydney University, Sydney 2751, Australia; 4 Centre for Mental Health, Swinburne University of Technology, Melbourne 3122, Australia; 5 Departments of Medical Genetics, Psychiatry, Physiology & Pharmacology, and Community Health Sciences, University of Calgary, Calgary, AB T2N 1N4, Canada; 6 Mental Health, St Vincent’s Hospital Melbourne, Melbourne 3065, Australia; 7 Department of Nutrition, Dietetics and Food, Monash University, Melbourne 3168, Australia; 8 Brain Park, Turner Institute of Brain and Mind, Monash University, Melbourne 3800, Australia

**Keywords:** anterior cingulate, GABA, generalised anxiety disorder, kava, magnetic resonance spectroscopy

## Abstract

Generalised Anxiety Disorder (GAD) is a prevalent, chronic mental health disorder. The measurement of regional brain gamma-aminobutyric acid (GABA) offers insight into its role in anxiety and is a potential biomarker for treatment response. Research literature suggests *Piper methysticum* (Kava) is efficacious as an anxiety treatment, but no study has assessed its effects on central GABA levels. This study investigated dorsal anterior cingulate (dACC) GABA levels in 37 adult participants with GAD. GABA was measured using proton magnetic resonance spectroscopy (^1^H-MRS) at baseline and following an eight-week administration of Kava (standardised to 120 mg kavalactones twice daily) (*n* = 20) or placebo (*n* = 17). This study was part of the Kava for the Treatment of GAD (KGAD; ClinicalTrials.gov: NCT02219880), a 16-week intervention study. Compared with the placebo group, the Kava group had a significant reduction in dACC GABA (*p* = 0.049) at eight weeks. Baseline anxiety scores on the HAM-A were positively correlated with GABA levels but were not significantly related to treatment. Central GABA reductions following Kava treatment may signal an inhibitory effect, which, if considered efficacious, suggests that GABA levels are modulated by Kava, independent of reported anxiety symptoms. dACC GABA patterns suggest a functional role of higher levels in clinical anxiety but warrants further research for symptom benefit. Findings suggest that dACC GABA levels previously un-examined in GAD could serve as a biomarker for diagnosis and treatment response.

## 1. Introduction

Generalised Anxiety Disorder (GAD) is a clinically challenging, prevalent, and chronic affective disorder associated with functional disablement, frequent comorbidities, and high psychological distress [1,2]. It is characterised by the two leading cognitive symptoms of persistent worry and anticipatory anxiety [3]. GAD prevalence and severity may be underreported, due in part to low diagnostic reliability [4,5,6,7]. Current stepped-care treatment for GAD typically involves antidepressant and benzodiazepine pharmacotherapies as well as cognitive behavioural therapy (CBT) [8,9,10]. These approaches, however, provide modest clinical effect and have limited utility for many patients [11,12,13,14,15,16]. Moreover, novel drug development has not progressed for two decades [6,17,18,19,20,21], leading many consumers to utilise anxiolytic phytomedicines [22,23].

GAD remains the most understudied of the anxiety disorders [6,24]. One promising avenue to advance GAD diagnosis and the assessment of treatment efficacy is to investigate the neuroimaging biomarkers [25,26,27,28] that may offer insight into the mechanisms of anxiety; however, the extent to which brain metabolic processes are instrumental in pathology remains unclear.

### 1.1. Gamma-Aminobutyric Acid (GABA) and Quantification in the CNS

GABA (C4H9NO2C4) is a non-standard amino acid that acts as the major inhibitory neurotransmitter [29,30]. GABA is distributed throughout the brain but is highly concentrated in cortical and limbic areas associated with ‘anxiety circuitry’ [31,32]. In these regions, multi-modal neuroimaging studies demonstrate a trend of emotion task-dependent stronger negative BOLD signal observed with a higher resting state GABA [33,34,35].

In pre-clinical rodent models, high anxiety-behaviour mice exhibit higher levels of amygdala GABA and a greater expression of GABA pathway components GAD65 and GAD67 than normal anxiety-behaviour mice [36]. Clinical studies place GABA in a central role in neuropathology across anxiety states and disorders [15,32,34,37,38,39], whereby elevated GABA is associated with anxiety cognitions in healthy samples in the ventral-medial prefrontal cortex (vmPFC) [40] and the medial pre-frontal cortex in post-traumatic stress disorder (PTSD) [41]. However, in PTSD, GABA levels are *reduced* compared to controls in the lateral temporal lobe, ACC, insula, and parieto-occipital cortices, but no differences are observed in the dACC region [42,43]. In panic disorder, GABA is reduced in the occipital and ACC regions and the basal ganglia [44,45], but it is not different in the pre-frontal cortex [46]. Social Anxiety Disorder (SAD) research suggests reduced thalamic GABA levels but no other regional differences compared with controls [47]. Taken together, the evidence suggests that GABA varies by region and disorder, but no research has yet directly assessed regional levels in GAD adults compared with healthy controls. In GAD intervention research, other metabolites such as n-acetyl aspartate following riluzole or paroxetine administration [48,49,50] have been investigated, but no study has thus far assessed GABA levels as a product of anxiolytic treatment.

### 1.2. The Dorsal Anterior Cingulate Cortex (dACC) as a Region of Interest

The ACC has a central role in the organisation of affective and cognitive information that underpin anxiety states via its connectivity to prefrontal-cortical, lower limbic, and hippocampal regions [51,52,53,54,55]. Sub-regions of the ACC are implicated in GAD symptomatology—notably, anticipatory anxiety and negative bias cognitions [56,57,58,59]. Structural and functional studies support a rostral/affective and a dorsal/cognitive division of ACC sub-regions, with a heterogeneous and integrative role of the latter in the cognitive components of emotional processing [51,53,60,61,62].

This dorsal region comprises a fear network along with the ventromedial prefrontal cortex and amygdala [63,64], with GABA levels in this region contributing to the maintenance of anxiety cognition–emotion fear responses and reducing fear extinction [61,62], suggesting that the cognitive control of worry thoughts is a hallmark of GAD. Yet, despite the evidence that ACC sub-regions are a significant predictor in intervention studies—at least in structural and functional studies [26,64]—it has received little focus in terms of metabolic data quantification to establish patterns in GAD symptomology, and for these reasons, the dACC is of particular interest in this investigation.

### 1.3. Kava for the Treatment of GAD Symptoms

The phytomedicine *Piper methysticum* (Kava) holds a compelling evidence base for the alleviation of anxiety symptoms [65,66,67,68], and it is a popular consumer treatment with over 1.2 million Kava extract tablets prescribed or purchased annually in Australia [69]. The water extraction method is the only allowable process in Australia, and tableted Kava products are typically standardised to 30% kavalactones, constituting quantities of 80 to 250 mg.

The Kava plant is a perennial shrub of the pepper family *Piperaceae*, and it is native to the South Pacific, where the rootstock lipid resin has been used for millennia in traditional medicine for its anxiolytic, nootropic, neuroprotective, nociceptive, and anti-dysphoria effects [70,71,72,73]. The chief bioactive constituents are kavalactones, six of which comprise the majority of Kava’s pharmacodynamic effects: dihydrokavain/dihydrokawain, kavain/kawain, dihydromethysticin, methysticin, yangonin, and demethoxyyangonin, in order of typical proportion [74,75,76,77].

The proposed mechanism of the *GABAergic* effect occurs through the positive modulation of multiple benzodiazepine binding sites, including GABA-A and GABA-B from kavain, yangonin, dehydromethysticin, and desmethoxyyangonin, through both enhanced ligand displacement and binding [75,78,79,80]. Pre-clinical studies also suggest mechanisms occurring within the GABA metabolic shunt, such as the modulation of the calcium ion channel blockade of the monoamine oxidase-B receptor substrate, thereby inhibiting glutamate and promoting GABA synthesis [70,81,82,83].

Previous Kava investigations in anxiety report significant improvements on anxiety scales, such as the HAM-A [22,84,85]. However, only one study has investigated the modulation of brain markers. In an electroencephalography (EEG) resting state study [86], acute doses of kavain (200, 400, 600 mg), placebo, and 30 mg of clobazam were administered to a healthy sample (*n* = 15). Significant dose-dependent increases were observed in frontal lobe delta, theta, and alpha 1 activity in the frontal lobe and, together with improvements to mood and wellbeing measures, were reported in the kavain group. The psychotropic effects and topographic pattern differed from the benzodiazepine, particularly with 200 mg, where benefits to mood measures were reported, but sedation was reported at higher doses. The data are consistent with pre-clinical models on kavain as a positive allosteric modulator of the GABA-A receptor outside the typical benzodiazepine site binding [75]. Data from this study contribute to the evidence of unique GABAergic action with kavalactones, supported by pre-clinical tissue studies. This single study also highlights the need to examine Kava effects via other neuroimaging modalities to better understand its neurobiological mechanisms.

To this end, this study aimed to investigate changes to GABA levels in the dorsal ACC in a GAD sample following the daily ingestion of Kava extract for eight weeks in comparison with a placebo group. This study also aimed to assess the relationship between GABA levels and changes to reported anxiety symptoms as a product of Kava consumption over the same period. It was expected that dACC GABA levels would decrease after Kava treatment and a reduction to anxiety symptoms would also be observed in comparison with the placebo group.

## 2. Methods and Materials

### 2.1. Design

This neuroimaging sub-study was conducted as part of the Kava for the Treatment of Generalised Anxiety Disorder clinical trial (KGAD) [87], a double-blinded, placebo-controlled investigation of 16-week’s administration of Kava in individuals with GAD. The sub-study’s eight-week duration was selected based on kava pharmacodynamics in both pre-clinical and clinical efficacy studies. The KGAD trial was registered via ClinicalTrials.gov (NCT02219880), with approval from the University of Melbourne/Alfred Hospital, the University of Queensland, and Swinburne University’s Human Research Ethics Committee. The clinical trial was conducted in accordance with the Declaration of Helsinki.

### 2.2. Participants

Participants involved in the neuroimaging component of the study were adults aged 18–65 years with Generalized Anxiety Disorder; *n* = 37 (male *n* = 18). Screening and eligibility criteria can be found in both the protocol and main outcome papers for the KGAD study [87,88] and are summarised in Appendix A. All enrolled participants received a bursary of $200 to cover expenses. Written and informed consent was collected from all participants before comprehensive screening for eligibility.

### 2.3. Sample Size

With an a priori population sample size of N = 40 (*n* = 20 per group), a 95% confidence interval, a 5% margin of error (for a standard alpha of 0.05), and a small-to-medium effect size (confidence interval reporting was the chosen parameter), a minimum total sample size of *n* = 37 was determined using the G.Power calculation program [89].

### 2.4. Measures

#### 2.4.1. Screening and Eligibility

The Mini-International Neuropsychiatric Interview (MINI 6.0) [90] was used to confirm the presence of current GAD according to the Diagnostic and Statistical Manual of Mental Disorders (5th ed.; DSM-5) [91], as well as identify secondary anxiety disorders and major depressive disorder (MDD) and the exclusion of other disorders. To ensure current GAD, moderate levels of anxiety (minimum 18 score) were required for enrolment to the study, assessed via the Hamilton Anxiety Rating Scale (HAM-A) [92]. The Montgomery–Asberg Depression Rating Scale (MADRS) [93] was utilised at both time points in the GAD group to ensure that symptoms of depression were not primary upon enrolment nor emergent following treatment (maximum score 18). The Structured Interview Guide for the MADRS was administered using a standardised line of questioning to ensure reliability and consistency (SIGMA) [94].

#### 2.4.2. Assessment of Comorbidity in GAD

Comorbidity (anxiety disorder or major depression) is not typically accounted for in neuroimaging studies with GAD samples. The prevalence of comorbidity in GAD is higher compared with other affective disorders, amplifying impairment [95,96]. Allowable secondary conditions were SAD, panic disorder with/without agoraphobia, and phobic disorder. MDD history was allowable if no episodes had occurred in the previous three years. This approach considers the likelihood of overlap in diagnoses in GAD where symptoms might be best explained by comorbidities such as MDD. Additional comorbid conditions may imply an increased severity of symptoms where higher anxiety measure scores may be observed. For this reason, a comorbid presence was either defined as a separate GAD group or used as a covariate in regression models.

#### 2.4.3. Assessment of Anxiety

The HAM-A is a 14-item 5-point Likert scale to quantify the severity of anxiety symptomatology. The Structured Interview version (SIGH-A) was used in this study to ensure a standardised questioning format in the assessment of anxiety levels at baseline and eight-week time points. The reliability and validity of the SIGH-A was assessed to be moderate to high [97].

### 2.5. H-MRS Protocol

A 3T Siemens TIM Trio magnetic resonance imaging system (Erlangen, Germany) with a 32-channel head coil housed at Swinburne Neuroimaging Centre (Hawthorn, Australia) was used for collecting ^1^H-MRS and T1-weighted structural imaging data. T1-weighted images were acquired for the localisation of the MRS voxel at dorsal anterior cingulate cortex (high-resolution 1 mm^3^, 176 slices, voxel resolution = 1.0 × 1.0 × 7.0 mm^3^, TR = 1900 ms, TE = 2.52 ms, flip angle = 9°, field of view 256 × 256 mm, orientation sagittal, acquisition time = ~3 min).

GABA quantification was conducted using Mescher–Garwood algorithm (MEGA) [98] in a Point RESolved Spectroscopy sequence (MEGA-PRESS), which is sensitive to GABA by editing the J-coupling between GABA-3 (peak at 3.01 ppm) and GABA-4 (peak at 1.89 ppm). The details of the sequences are as follows: TE = 68, TR = 2000, suppression freq. = 1.95 ppm, Ave = 64, ~5 min, editing pulse at 1.95 ppm (edit-on) interleaved with scans with a pulse at 7.5 ppm (edit-off). The water signal (unsuppressed signal) was acquired at the identical location with 16 averages for two minutes’ duration. Shimming was automatic and manual until the linewidth was less than 20 Hz for the 80/68 TE PRESS and MEGA-PRESS sequences.

#### 2.5.1. Voxel Placement and Check

**Figure 1:**
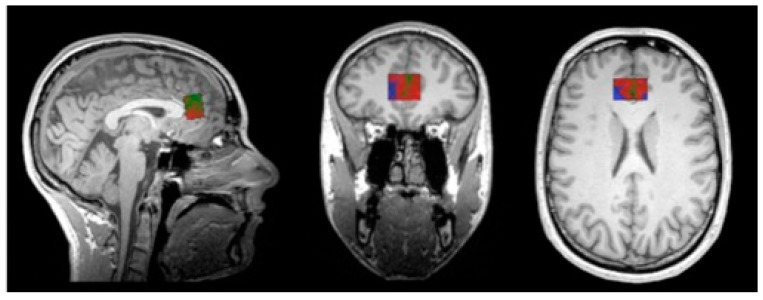
An example of tissue segmentation at individual space to segment grey matter (GM), white matter (WM), and CSF. The volumetric proportion (percentage) of each tissue type within the MRS voxel was further calculated for a partial volume correction check.

**Figure 2:**
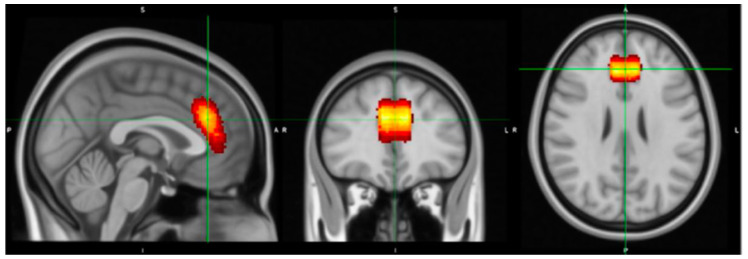
The averaged voxel location for MRS for all scans transferred into MNI space; the target region is the dorsal subregion of the anterior cingulate cortex (dACC).

The MRS voxel was 25 × 25 × 15 mm and covered the dACC. An in-house MATLAB (R2013b) script was applied to build a voxel image based on the location information on the head of MRS data. This voxel image indicates the location of the MRS voxel, when overlapping on the corresponding structural image at the same scan session. To check whether the location of the MRS voxel covered the target region, i.e., dACC at coordinates [0, 34, 26] in MNI (standard) space, individual structural images were co-registered to a MNI template, and the same transform matrix was applied on the corresponding voxel images. Finally, tissue segmentation was conducted at individual space to segment grey matter (GM), white matter (WM), and CSF. The volumetric proportion (percentage) of each tissue type within the MRS voxel was further calculated for partial volume correction and quality check (refer to Figure 1). All individual voxel images were summarised and plotted on top of an MNI template using MRIcron [99], visible in Figure 2, where the dACC was well covered.

#### 2.5.2. GABA Analysis

The difference spectrum (edit-on versus edit-off) was used to quantify the concentration of metabolites, including GABA. Fit checks were performed during preprocessing using GANNET, which utilises a peak fitting/integration method and applies a stricter model in its Fit Error quality control, aiding the removal of spurious cases [100]. Following vetting of the data against quality check parameters (SNR > 5, SD < 20%, FWHM < 0.15), the voxel location was also visually inspected by overlapping the reconstructed voxel image and the T1-weighted MR image using in-house script and the MRIcron program. The LCModel toolbox (version 6.1) [101,102] is a widely used program for ^1^H-MRS analyses, and a standard GABA basis set, matching the imaging protocol, was used for model fittings.

Partial volume correction (e.g., voxel segmentation) is a crucial component of the imaging pipeline when assessing voxel metabolite levels to prevent the inflation of quantification data that come from variances in grey and white matter [103,104,105,106]. As the concentration of GABA in grey matter is substantially higher than in white matter, further partial volume effects were corrected approximately by considering the grey matter volume ratio of the voxel of interest [107]. Briefly, tissue segmentation was conducted at a whole brain level by SPM12; then, the reconstructed MRS voxel (detailed in Section 2.5.1) were overlapped on tissue maps to determine the volume of grey matter, white matter, and CSF. The corrected GABA concentration was converted using the equation below, where GMV, WMV, and CSF represents the volume of grey matter, white matter, and CSF.

$${GABA}_{corrected}={GABA}_{LCModel}÷\frac{GMV}{GMV+WMV+CSFV}\times 100\%$$

### 2.6. Treatment Handling

Procedures relating to treatment randomisation, the handling of treatment-related adverse events, and liver function assessment, as well as treatment compliance, are detailed in the KGAD study protocol and main outcome papers.

### 2.7. Statistical Analyses

Data were analysed using the IBM Statistical Package for Social Sciences software (SPSS; v.22, Armonk, NY, USA). All relevant demographic data were assessed at baseline treatment group differences via simple significance testing (*t*-tests and non-parametric tests were used when parametric criteria were not met), following quality checks for outliers and missing or incorrect data. Volumetric data, GABA QC variables, and GABA concentration results were reported separately by means and SDs for the variable of interest. It is feasible that age and sex-related differences would be observable in the samples tested in this study, potentially affecting results if not accounted for. For this reason, age and sex were employed in all group-based analyses to best delineate treatment group-based differences in function and GABA. An initial correlation test was performed to assess the relationship between GABA concentration levels and anxiety baseline data with covariates of sex, age, and comorbidity.

Intervention analyses were performed using a custom main effects format for GABA outcomes, with the main outcome reported from the interaction of *treatment x time* on GABA concentration and a linear mixed model format for *treatment x time x GABA* significance on anxiety symptoms change. Confidence intervals were reported to establish parameter estimates, with a proportion of variance (reported in the text as a percent) for significant predictors. Statistical findings were considered significant if the probability value *p* ≤ 0.05.

## 3. Procedure

Participants attended a screening session at the study site (Swinburne University of Technology, Melbourne) to assess study eligibility, followed by a baseline session, a supply of study treatment (240 mg daily tablets of kava or placebo), and a follow-up at eight weeks. Sociodemographic information included age in years, sex (male/female), BMI, medical conditions, alcohol consumption per week, allowable medication use (prophylactics), and eligible vitamins and supplements as determined by the protocol for the KGAD study [87]. Blood samples were collected at screening and follow up to assess liver function following overnight fasting, without alcohol or caffeine in the previous 24 h period. A standardised breakfast was provided followed by demographic data collection and psychiatric assessments. The scan was conducted in the same building one hour following arrival for the testing sessions. Processes, including the time of visit and scheduled scan, were replicated at the second time point (eight weeks).

## 4. Results

### 4.1. Description of the Study Population

**Figure 3:**
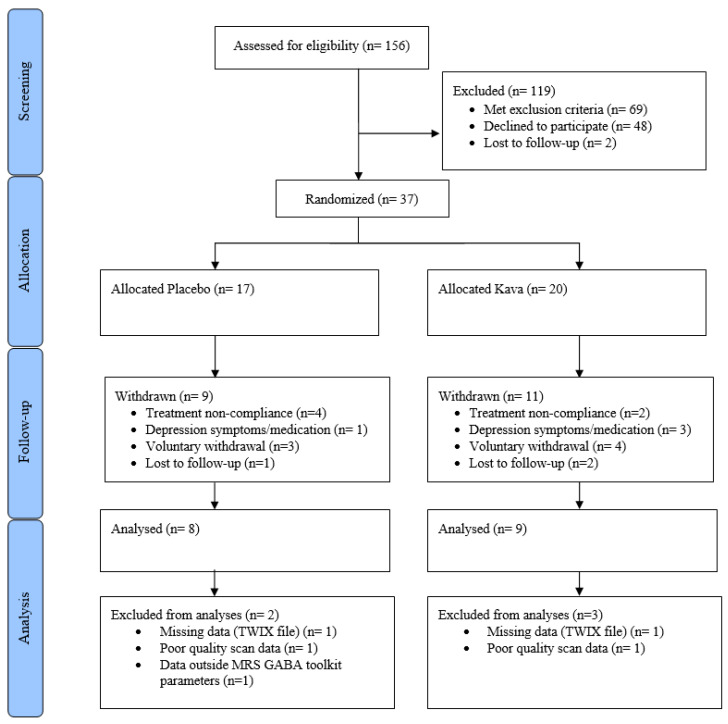
CONSORT diagram for intervention.

The *CONSORT diagram* in Figure 3 illustrates the study processes as well as participant numbers at screening, allocation, follow-up, and analysis. Demographic statistics overall and per treatment groups, including HAM-A scores and GABA data, are reported in Table 1. Treatment groups at baseline showed no significant differences in demographic data including age, sex, years of education, medication use, and caffeine or alcohol use. Baseline HAM-A scores for anxiety and the MADRS for depression symptoms were not significantly different between treatment groups (*p* = 0.714; *p* = 0.456, respectively).

### 4.2. Adverse Events

Adverse events reported during the study enrolment were not significantly different between participants in the kava and placebo groups (*p* = 0.508). The active treatment *P. methysticum* extract was well tolerated, with one participant reporting an adverse event of dizziness and a headache of mild severity with a probable association to kava. There were no serious adverse events reported in this study.

### 4.3. Withdrawals

The attrition of the sample size following the baseline visit was 46% (*n* = 17) and was not significantly different between the two groups (*p* = 0.482) for the following reasons and frequencies: non-compliance with study protocol (*n* = 6), lost to follow-up (*n* = 3), personal reasons/unspecified (*n* = 5), psychiatric status change (depression symptoms worsening, *n* = 2), medication status change (antidepressant course, *n* = 2), and pregnancy (*n* = 1).

### 4.4. Structural Data and Metabolite Quantification

The volumetric analysis showed no significant treatment group differences. The only quality parameter to display significant group differences was FWHM, *t*(37)2.29, *p* = 0.025, which was higher in the kava group (m 0.09, SD 0.05) than placebo (m 0.06, 0.02), meaning a better quality signal for the placebo group.

#### 4.4.1. GABA Concentration Levels and HAMA at Baseline

The *t*-test for the treatment group difference on anxiety levels via the HAMA at baseline was not significant (*p* = 0.932). Partial correlation revealed a significant moderate positive relationship between HAMA scores and GABA concentration level, *r*(24) = 0.40, *p* = 0.05.

#### 4.4.2. GABA Concentration Level Changes as a Function of the Eight-Week Kava Treatment

**Figure 4:**
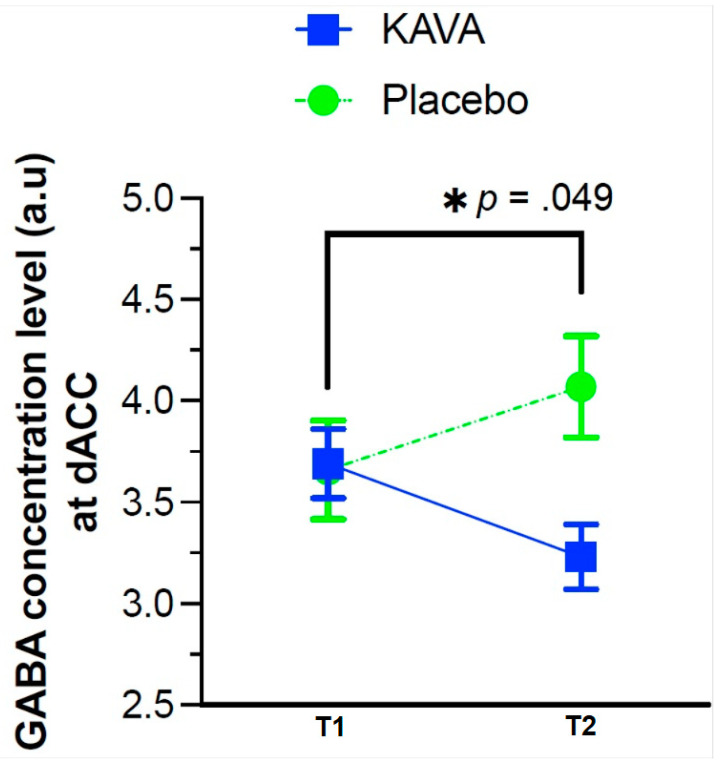
LCModel GABA means per group, baseline (T1) to eight weeks (T2).

The partial volume-corrected GABA concentration in the grey matter model showed a significant treatment by time interaction, *F*(1, 21) = 4.36, *p* = 0.049. The model is shown in Table 2. Pairwise comparisons at the second time point indicated that the kava group was 1.06 units lower than the placebo group, and this was statistically significant (*p* = 0.008), 95% CI [−1.83, −0.28]. Refer to Figure 4.

## 5. Discussion

The purpose of the study was to investigate GABA concentration in the dorsal ACC in adults with diagnosed GAD and to assess whether these levels were modified by Kava. A significant difference in GABA concentration was observed in the Kava group at eight weeks compared with the placebo group, showing a reduction in GABA concentration levels in the ROI.

The study operated as a ‘proof of concept’ investigation, both in establishing the measurement of GABA in GAD and in evidencing neural mechanisms amenable to Kava. It addressed these aims through assessing GAD symptoms and biological outcomes before examining the role of Kava on both GABA levels and anxiety symptoms.

This study found that baseline anxiety levels were positively associated with GABA levels in the dACC but that a daily dose of 240 mg *P. methysticum* extract for eight weeks was not successful in reducing anxiety symptomatology at the eight-week time point. The overarching clinical trial for which this study was conducted similarly found no anxiety improvements in the Kava group over the 16-week treatment period [88]. These findings contribute evidence for a lack of efficacy of the particular Kava extract in GAD, which is in contrast to earlier studies, reviews, and meta-analyses suggesting otherwise [22,108,109]. A caveat may be the small–moderate effect sizes in intervention efficacy studies [10], where many findings were also equivocal, supporting the observation in the literature that GAD is a clinically challenging disorder to treat.

Despite these findings, results suggest that Kava modifies brain GABA levels, as GABA level reductions were observed in the Kava group in the dACC region compared with the placebo group. This reduction could represent a ‘normalisation’ in GABA levels, which is reflected in the handful of studies in the area that show that raised GABA is linked to anxiety levels in healthy samples, as well as elevations in PTSD compared with healthy controls [41,42]. Overall, the evidence is not sufficient to conclude trends in the dorsal ACC or within GAD groups.

Given the bioactive constituent profile of the extract used in this study and the GABAergic mechanisms exerted by kavain, dihydrokavain, and methysticin in particular, it is feasible that the observed effects have occurred via enhancement to particular GABA-A receptor subtypes in the ROI through direct ligand-binding enhancement or indirectly via reductions to thromboxane A (2), which antagonises GABA-A receptor functions. It is also likely that modulations have occurred elsewhere in the shunt, such as the excitatory glutaminergic corollary through calcium ion channel blockades via the MAO-B receptor substrate, and there is increasing evidence of glutaminergic modulation in preclinical research [110]. The precise mechanisms, and the relationship to GABA levels, are yet to be understood through pre-clinical studies, but modulation within the shunt could improve the physiology of the GABA substrate, shown through reduction to GABA levels in the ROI.

### 5.1. Limitations in This Current Study

Importantly, a sample size attrition of 46% from baseline to the second time point (*n* = 8 kava; *n* = 9 placebo), resulting in the reduced power of this study overall and the reduced accuracy of predictor effects, will impact the interpretation of the results. A borderline *p*-value for the interaction terms in the model may reflect this attrition. The reporting of confidence intervals, illustrating appropriate variance ranges, may have added some support to a significant result. Although the use of linear mixed modelling is partly protective against such impacts, the results need to be considered in this context.

Secondly, GABA concentration levels in the brain derived from spectral data requires high amounts of data modelling to expose GABA peaks, and there is debate on the optimal methods for sequencing, spectral analysis, quality parameters, or data modelling of ‘contaminants’ such as macromolecules. All magnify the risk of measurement errors, among other methodological issues [111,112,113]. However, an advantage was the MEGA-PRESS protocol, which is able to derive metabolite data separate from macromolecules, and the use of GANNET in the processing pipeline. Furthermore, LCModel’s toolbox-specific quality parameters could identify technical factors affecting the analysis of GABA levels. Despite removing scans of spurious quality, group differences were still observed for these variables in this current study. FWHM, as a measure of linewidth in the spectrum quantifying the signal decay rate in the time-domain as a result of shimming, was significantly different between the treatment groups. It is possible that the methods applied within the toolbox have influenced the observed GABA concentration, thereby requiring caution in interpreting the results and subsequent conclusions. However, the baseline GABA concentration was not related to FWHM, so the different quality parameters had limited impact on the reported findings.

Thirdly, scan novelty may also be an influencing factor in participants with clinical anxiety. This current study did not collect data as to whether participants were familiar with MRI scan environments. A ‘sham’ scan session for each participant might have reduced this confound.

For these reasons, this study’s novel findings should be considered pilot data at best, offering a novel insight into the pattern of GABA levels in a GAD as a product of Kava, as well as an example of greatly needed biomarker research for the benefit of clinical anxiety.

### 5.2. Future Research Directions

The findings in this study between Kava treatment, anxiety symptoms, and GABA levels warrant further examination. Given the role the dACC may play in hallmark anxiety cognition symptoms in GAD, as well as evidence of kava efficacy in the literature, it would be of value to examine whether kava-based modulations were observed on selected HAM-A items (such as cognitive over somatic symptoms), which might clarify the pattern observed in the ROI. Similarly, a division of higher anxiety and low anxiety may reveal different Kava responses. Secondly, beyond the modulation of GABA by Kava, given the likely mechanisms in the GABA metabolic shunt of Kava in preclinical studies on NMDA receptors, an examination of glutamate levels may also reveal Kava mechanisms beyond GABA, supporting recent preclinical research and offering potential avenues for future therapeutic approaches.

This current study was valuable for understanding the mechanisms of Kava in GAD, and future research should extend Kava dosing in other anxiety cohorts and healthy controls and assess patterns of GABA levels in comparison with GAD. This could include major depressive disorder or state-based anxiety disorders, such as phobias and panic disorder, to delineate differences in ROI GABA levels and determine the relationship to anxiety symptoms.

There remains a shortfall in brain biomarker research in anxiety disorders, especially GAD. Emerging approaches now exist to guide the identified need, such as the Research Domain Criteria (RDoC) framework [114,115], which was founded on the premise that it is insufficient to assess symptoms alone to treat (or diagnose) GAD, yet there is insufficient data serving as biomarker evidence. This current study serves to address this shortfall. Biomarker treatment approaches assert that biological patterns may not fit with current diagnostic categories, signalling a vital need for research that clarifies neurobiological marker roles in symptom maintenance and response patterns with treatment [115,116,117,118]. These markers also have potential to identify disorders before the onset of anxiety symptoms and to identify variants or clinically meaningful subsets [18,119,120]. What is measured as biomarkers in neuroimaging is arguably closer to the exact substrates that underpin disorders, and we are therefore able to more precisely gauge the relationship between biomarkers and clinical endpoints for the individual [18,25,115,121].

This current study showed that GABA metabolic data were successful in producing differences that could be utilised as GAD biomarkers, with some considerations. The assessment of the neurobiological effects of Kava is novel in GAD samples and shows how our methodology could be applied to other anxiolytic phytomedicines. Data from MRS GABA can contribute to an integrative efficacy model of imaging modalities and psychological measures for future efficacy studies of biological substrates. More work is needed in the establishing of biomarkers for clinical benefit, but there is a strong translational rationale for the investigation of novel anxiolytics like Kava as GAD treatment, as demonstrated in this current study.

## 6. Conclusions

In order to elucidate the role of GABA or its metabolic components either as a predisposing, preventative, or therapeutic utility, it is important to quantify GABAergic processes. This research represents the first study to assess brain GABA concentration levels in adults with GAD, as well as the first to assess modulations following a treatment intervention using Kava. This study demonstrated that GABA levels are modulated via Kava treatment, and regional brain GABA concentration levels could be linked to anxiety symptoms in GAD. The findings also indicate that GABA levels can change without concomitant changes in anxiety symptoms, suggesting that symptom profiles in GAD is complex and multi-factorial. Further studies quantifying brain anxiety markers over time, controlling for other associated variables, and the use of a benzodiazepine comparator could clarify the role of GABA as a marker of anxiety in brain regions associated with cognitive anxiety symptoms. It is also vital for future studies to determine levels of GABA in brain regions using wider healthy cohorts.

## Figures and Tables

**Table 1:** Demographics and GABA data for treatment groups and time points.

Demographics *Mean (S.D.), n or % where relevant*	Baseline Whole Group	Baseline Placebo	Baseline Kava	Tmt *p*-Value *	Week 8 Whole Group	Week 8 Placebo	Week 8 Kava
Participants (n)	37	17 (9 male)	20 (11 male)	0.653	20	9	11
Age	36.16 (13.09)	36.06 (11.31)	36.25 (14.73)	0.965	-	-	-
Education (years)	17.65 (3.91)	18.59 (4.70)	16.85 (2.98)	0.181	-	-	-
*Psychiatric*							
Comorbid condition	21	12	9	0.110	-	-	-
Comorbid: SAD	11	6	5	0.395	-	-	-
Comorbid: PD	9	7	2	0.034 *	-	-	-
Comorbid: AGO	17	9	8	0.324	-	-	-
Comorbid: PTSD	1	1	0	0.460	-	-	-
Comorbid: MDD	20	9	11	0.581	-	-	-
HAM-A ^a^	23.05 (3.60)	23.29 (4.19)	22.85 (3.10)	0.714	15.55 (5.69)	14.00 (6.20)	16.82 (5.17)
MADRS ^b^	13.59 (2.99)	14.00 (2.91)	13.25 (3.09)	0.456	10.38 (5.30)	10.67 (4.82)	10.17 (5.84)
*Medical*							
Medications	15	7	6	0.357	-	-	-
Supplements	9	2	7	0.103	-	-	-
*Substance*							
Caffeine (mg/daily)	135.54 (127.64)	156.47 (132.96)	117.75 (123.51)	0.365	100.55 (83.71)	107.27 (68.93)	94.50 (98.52)
Alcohol (SD/weekly)	2.86 (3.08)	1.88 (2.02)	3.70 (3.60)	0.073	4.10 (3.65)	1.67 (1.50)	6.09 (3.73)
*Volumetric*							
dACC GM (%, mm^3^)	60.29 (13.20)	59.41 (12.22)	61.05 (14.25)	0.712	58.35 (12.33)	53.11 (15.89)	62.64 (6.45)
dACC WM (%, mm^3^)	23.48 (11.46)	24.58 (11.86)	22.55 (11.34)	0.597	23.40 (10.78)	27.67 (13.90)	19.91 (6.02)
dACC CSF (%, mm^3^)	15.57 (5.04)	14.53 (3.93)	16.47 (5.77)	0.249	18.01 (6.18)	19.44 (4.56)	16.84 (7.26)
*GABA toolkit*							
LCModel GABA/GM	3.67 (0.88)	3.66 (1.01)	3.69 (0.77)	0.932	3.59 (0.74)	4.07 (0.75)	3.23 (0.53)
LCModel SD (%)	9.39 (2.30)	9.47 (1.58)	9.31 (2.83)	0.843	9.50 (2.68)	8.89 (0.78)	10.00 (3.55)
LCModel SNR	9.00 (2.58)	9.70 (2.26)	8.37 (2.75)	0.123	9.05 (2.42)	9.44 (2.13)	8.73 (2.69)
LCModel FWHM	0.08 (0.04)	0.06 (0.02)	0.09 (0.05)	0.025 *	0.07 (0.03)	0.06 (0.02)	0.08 (0.04)

* Significance values derived from independent sample *t*-tests or Chi Square tests where appropriate and significant, where *p* < 0.05; ^a^ Hamilton Anxiety Rating Scale; ^b^ Montgomery–Asberg Scale of Depression; AGO—Agoraphobia; MDD—Major depressive disorder; PD—panic disorder; PTSD—Post-traumatic stress disorder; SAD—social anxiety disorder; SD—standard drink/10 g of alcohol; CSF—cerebrospinal fluid; df—degrees of freedom; dACC—dorsal anterior cingulate cortex; GM—grey matter; Fit Err—Fit error %; FWHM—full width at half-maximum; GABA—gamma amino-butyric acid; ROI—Region of interest, SNR—signal–noise ratio; SD—standard deviation; Tmt—treatment (group difference); WM—White Matter.

**Table 2:** Corrected GABA concentration changes, baseline to eight weeks.

GABA Model	B/Estimate	95% CI	t	df	*p*-Value
Corrected GABA concentration					
Time	0.48	−0.09, 1.06	1.74	21	0.097 ^a^
Treatment	1.06	0.28, 1.83	2.75	46	0 0.008 *
Sex	−0.28	−0.78, 0.23	−1.14	21	0 0.268
Age	0.01	0.00, 0.03	1.58	19	0.129
Comorbid	0.13	−0.39, 0.65	0.53	24	0.603
Baseline alcohol	0.05	−0.04, 0.03	1.58	21	0.129
Time*Treatment	−0.87	−1.74, 0.00	−2.09	21	0 0.049 *

* Significant at *p* < 0.05; ^a^ Marginally significant at *p* = 0.05–0.10; B/coefficient unit decimal places are variable due to LCModel level ranges; df—degrees of freedom.

## Data Availability

Data were collected exclusively for the purpose of fulfilling the NHMRC grant research within a clinical group. Additionally, provision to third parties may not comply with Australian clinical trial grant mandates. To this end, the data from this study are not publicly available.

## Appendix A

Enrolment Criteria
**Generalised Anxiety Disorder Group**
Inclusion Criteria
- Meets the DSM-5 diagnostic criteria for generalised anxiety disorder based on structured interview MINI 6.0. Note that while the MINI 6.0 uses the DSM-IV criteria, the same criteria are used in the DSM-5.
- Presentation with anxiety (HAM-A ≥ 18) at the time of study entry - Presentation of mild depressive symptoms (MADRS ≤ 18) at the time of study entry
Exclusion Criteria
- Primary diagnosis other than GAD - Presentation of moderate to severe depressive symptoms (MADRS ≥ 18) at the time of study entry - Presentation of suicidal ideation (≥ 3 on MADRS suicidal thoughts domain) at the time of study entry - Current diagnosis of a psychotic disorder (bipolar disorder I, schizophrenia) on structured interview (MINI 6.0) - Current or past diagnosis of obsessive compulsive disorder or bipolar I or II disorders - Current substance/alcohol use disorder on structured interview (MINI 6.0) - Currently taking an antidepressant, mood stabiliser, antipsychotic, anticonvulsant, warfarin, or thyroxin, or regularly using a benzodiazepine or opioid-based analgesic (more than 2 days per week) - Current use of St John’s wort - Three or more failed trials of pharmacotherapy for the current GAD episode - Recently commenced psychotherapy (within four weeks of study entry) - Known or suspected clinically unstable systemic medical disorder - Pregnant or breastfeeding or trying to conceive - Not using a medically approved form of contraception (including abstinence) if female and of childbearing age - Unable to participate in all scheduled visits, treatment plan, tests, and other trial procedures according to the protocol
Imaging eligibility criteria—exclusions
- Left-handed - Pacemaker - Infusion pumps - Aneurysm clips - Metal prostheses - Metal joints - Metal rods - Metal plates - Metal staples - Non-removable body piercings - Persons who have worked as welders or may have metal splinters in their body - Females: taking hormone-modulating contraception including oral contraceptive pill or implants - Females: irregular menstrual cycle in order to ascertain days 1–10 for the conducting of scans
